# Antidiabetic effects of fennel leaf aqueous extract in alloxan-induced diabetic rats

**DOI:** 10.1186/s40780-025-00458-x

**Published:** 2025-06-16

**Authors:** Mahdi Noureddini, Maryam Akbari, Zeinab Vahidinia, Samaneh Sadat Alavi, Majid Nejati, Mohammad Ali Atlasi

**Affiliations:** 1 Physiology Research Center, Institute for Basic Sciences, Kashan University of Medical Sciences, Kashan, Iran; 2 Anatomical Sciences Research Center, Institute for Basic Sciences, Kashan University of Medical Sciences, Kashan, Iran; 3 Department of Anatomy, School of Medicine, Kashan University of Medical Sciences, Kashan, Iran

**Keywords:** Fennel leaf, Alloxan, Diabetes mellitus, Islets of Langerhans

## Abstract

**Background:**

Diabetes Mellitus is a common chronic metabolic disease in the world population. There is evidences on the anti-hyperglycemic effects of different parts of fennel; however, the reports about antidiabetic activity of fennel leaves are not enough. In this experiment, effects of fennel leaf aqueous extract on biochemical alterations and the histopathology of the pancreas in alloxan induced diabetic rats were studied.

**Methods:**

Fifty adult male rats were divided into five groups: the non-diabetic and the diabetic control groups, and three diabetic groups treated with different doses of fennel leaf extract (50, 100 and 200 mg/kg/day). Blood glucose, body weight, serum insulin and C-peptide levels were determined. The pancreas histology was evaluated by preparation of paraffin sections. They were stained using hematoxylin and eosin stain. Morphometrically, the mean number and the area of the islets of Langerhans were measured.

**Results:**

Fennel leaf extract in different doses caused a reduction in blood glucose, and an increase in body weight, serum insulin and C-peptide. In diabetic treated rats, fennel leaf extract significantly increased the number and area of the Islets of Langerhans.

**Conclusions:**

Our results indicated the anti-hyperglycemic effects of fennel leaf extract and morphologic improvement of the pancreatic islets of Langerhans in alloxan induced diabetic rats.

## Introduction

Diabetes mellitus is a common chronic human disorder and is a globally important cause of morbidity and mortality [1]. Diabetes is estimated to cause one in nine deaths among adults aged 20–79 years[2] and its global prevalence is estimated at 529 million people living with diabetes worldwide [3]. Chronic hyperglycemia in diabetes mellitus leads to the development of micro- and macro vascular diseases which cause irreversible damage [4].The damage is primarily due to high oxidative stress, an increase in ROS levels and the activation of inflammatory pathways [5].

For a long time, medicinal plants have been used for the prevention and treatment of diseases. In most developing countries, plant drugs are the primary choice for health care needs [6]. The role of traditional medicine in the treatment of diabetes is attractive in scientific research [7–9]. Phenolic acids, flavonoids, and terpenoids are major antidiabetic components of traditional medicinal plants [10]. The hypoglycemic activity of these components is attributed to stimulating the secretion of insulin by the islets of Langerhans in the pancreas, the reduction of insulin resistance, the antioxidant activity, and the inhibition of α-glucosidase and α-amylase [9].

Fennel is an important medicinal and aromatic plant that is widely used in traditional medicine. Although it is observed that fennel is endemic to the Mediterranean, in other world regions such as Iran this plant wildly grows and is cultivated [11]. The essential oil extracted from fennel exhibited antibacterial, antifungal, antithrombotic, and anti-hirsutism activities [12, 13]. It has long been used for treating respiratory and gastrointestinal disorders [12]. In addition, hypoglycemic and antioxidant activities of the fennel have been reported [13, 14]. The leaves of fennel have been shown to contain a number of flavonoids, fixed oil, protein, and organic acids, which are responsible for antioxidant, and antidiabetic effects [9, 15]. A few reports have shown the effects of fennel leaf on blood glucose levels in streptozotocin induced diabetic rats [14]. In spite of all that, there are little evidence about biochemical and histopathological effects of fennel leaf in diabetes.

The aim of the present study is to evaluate the effects of aqueous extract of fennel leaves on blood glucose level and histopathology of pancreas in alloxan induced diabetic rats.

## Material and methods

### Extraction method

The fennel was purchased from a local herbal market in Kashan,Iran and approved by Kashan Botanical Garden (H.N: H.K.B.G.1065 GPS:33.96983629910757,51.477861799921016). After identifying the plant, its leaves was separated and dried in the shadow. Then, the ground fennel leaf was extracted using soxhlet extractor (aqueous). After evaporation and reduction of the solvent by a rotary evaporator, it was dried in an oven and refrigerated at 4ºC until use. *A total of 100 g of fennel leaves were used for extraction, yielding 8.75 g of extract*. All these processes were done in the Kashan Essence Research Center.

### Animals and study design

Fifty Sprague–Dawley male rats weighing 150–200 g were obtained from the laboratory animal house, Kashan University of Medical Sciences (KAUMS), Iran. Rats were maintained at 21–24 °C with a 40–60% relative humidity and 12 h light/dark cycle intervals with standard rat feed and water ad libitum. The ethical committee of KAUMS approved the experiment (ethical code: 1911).Animals were divided into five groups, 10 rats in each group. Groups I and II included non-diabetic and diabetic control rats respectively. Groups IIIa, IIIb, and IIIc were three groups for diabetes treatment. Diabetes was induced by a single intraperitoneal injection of alloxan at the dose of 170 mg/kg body weight. Alloxan solutions were freshly prepared and made by alloxan monohydrate (Sigma) dissolved in acetate buffer (pH 4.5). The rats fasted for 12 h before and after the injection. Six days following alloxan injection, blood glucose was measured. Rats with blood glucose levels 220–250 mg/dL were determined as diabetic animals [16]. Groups IIIa, IIIb, and IIIc consisted of diabetic rats that were treated with three different doses of fennel leaf aqueous extract orally (50, 100, and 200 mg/kg body weight respectively).

### Measurements of blood glucose and body weight

On days 0, 7, 14, 21, and 30 from the beginning of the experiment, body weight and blood glucose were recorded in each group. For the measurement of blood glucose, after overnight fasting, a sample of tail blood was drawn by introducing the needle into the distal end of the tail and blood glucose was measured with a blood glucose monitoring device[17].

### Measurements of blood insulin and C-peptide concentrations

After 30 days from the experiment, the animals from each group was anesthetized with ether, and blood samples were drawn from the heart. After centrifugation of the samples, serum was separated from the blood cells and maintained at −80 °C. Insulin(μIU/mL) and C-peptide**(**pg/ml) concentrations were measured in serum using the kit (Pars Azmoon Co, Tehran, Iran).

### Histopathological study

By the end of the experiment and after collection of blood samples from the heart, rats were sacrificed. The whole pancreas was removed, fixed in 10% buffered formalin, processed, and embedded into paraffin blocks. Paraffin Sects. 6 μm thick were stained with Hematoxylin and Eosin (H&E). The slides were studied under the light microscope (Nikon, Germany). By a camera connected to the microscope, the × 100, × 200 and × 400 images of the sections were prepared. For each Sect. 5 fields were selected and studied using a light microscope (Nikon, Germany). [18].Morphometrically analysis for the islets of Langerhans was done using imaging software using the DS camera Control Unit DS-L2. The mean number and areas of the islets and the mean number of cells in each islet are estimated.

### Statistical analysis

Data were analyzed and presented as means ± SD. Differences between data were analyzed using one-way ANOVA followed by Tukey’s post hoc test. *P* < 0.05 was considered significant.

## Results

### Effect of fennel leaf aqueous extract on blood glucose concentration

**Fig. 1:**
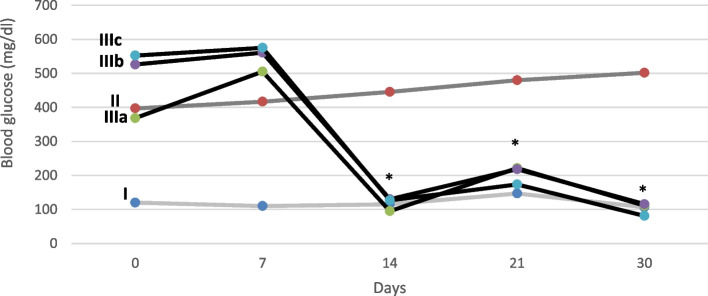
Blood glucose level on different days in five groups of the experiment. *P < 0.01

Blood glucose concentration increased significantly (*P* < 0.05) following alloxan injection compared with the control group during the experiment. The rats in groups IIIa, IIIb and IIIc that received doses of 50, 100 and 200 mg/kg fennel, respectively, showed a significant decrease (*P* < 0.01) compared with diabetic group II on days 14, 21, and 30 (Table 1; Fig. 1).

**Table 1:** Blood glucose concentration in different groups of the experiment

Experimental groups	Period of treatment				
	**0d**	**7d**	**14d**	**21d**	**30d**
**Group I** (Control)	120 ± 3.07	110 ± 1.65	115 ± 1.84	146.84 ± 4.42	106.65 ± 1.65
**Group II** (Diabetic)	397 ± 41.9	416.75 ± 50.04	445.5 ± 36.36	479.83 ± 59.23	501.83 ± 63.9
**Group IIIa** (Fennel 50 mg/kg BW)	368.43 ± 56.35	505.29 ± 64.16	95.14 ± 12.79*	221.57 ± 53.97*	111.14 ± 18.9*
Group**IIIb** (Fennel 100 mg/kg BW)	526 ± 68.3	561 ± 18.68	130 ± 32.04*	218.14 ± 67.41*	115.57 ± 28.51*
Group**IIIc** (Fennel 200 mg/kg BW)	552.5 ± 6.4	575.17 ± 31.26	126.73 ± 14*	173.5 ± 10.7*	81.17 ± 29.9*

^*^P < 0.01

### Effect of fennel leaf aqueous extract on the body weight

**Fig. 2:**
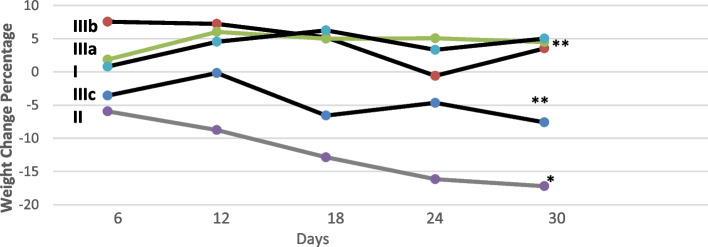
Percentage of body weight changes in different groups of the experiment. *P < 0.05, ** *P* < 0.01

After 30 days from the beginning of the experiment, rats in the diabetic group significantly lost their initial body weight (*P* < 0.05) as compared with the normal control group (Table 2; Figure 2).

**Table 2:** Percentage of body weight changes in different groups of the experiment

Experimental groups	Period of treatment					
	**0d**	**6d**	**12d**	**18d**	**24d**	**30d**
**Group I** (Control)	0	0.81 ± 1.3	4.55 ± 2.4	6.26 ± 2.80	3.33 ± 3.096	5.04 ± 2.38
**Group II** (Diabetic)	0	−5.937 ± 0.44	−8.75 ± 0.83	−12.86 ± 0.85	−16.15 ± 0.99	−17.20 ± 0.83*
**Group IIIa** (Fennel 50 mg/kg BW)	0	1.86 ± 0.53	6.04 ± 0.55	5.013 ± 0.77	5.08 ± 0.77	4.50 ± 0.55**
Group **IIIb** (Fennel 100 mg/kg BW)	0	7.57 ± 0.78	7.24 ± 0.59	5.20 ± 0.3	−0.59 ± 0.5	3.55 ± 0.59**
Group **IIIc** (Fennel 200 mg/kg BW)	0	−3.56 ± 0.31	−0.16 ± 0.59	−6.56 ± 0.77	−4.66 ± 0.79	−7.59 ± 0.59 **

^*^P < 0.05, ** *P* < 0.01

Following the administration of fennel leaf aqueous extract at 50 mg/kg and 100 mg/kg for 30 days increased their initial body weight to the same level as the normal control group (P < 0.01). After 30 days of treatment with 200 mg/kg of fennel leaf aqueous extract, body weight was significantly greater than that in the diabetic group (*P* < 0.01) and less than in IIIa, IIIb groups and I (*P* < 0.05).

### Effect of fennel leaf aqueous extract on serum insulin concentration

**Fig. 3:**
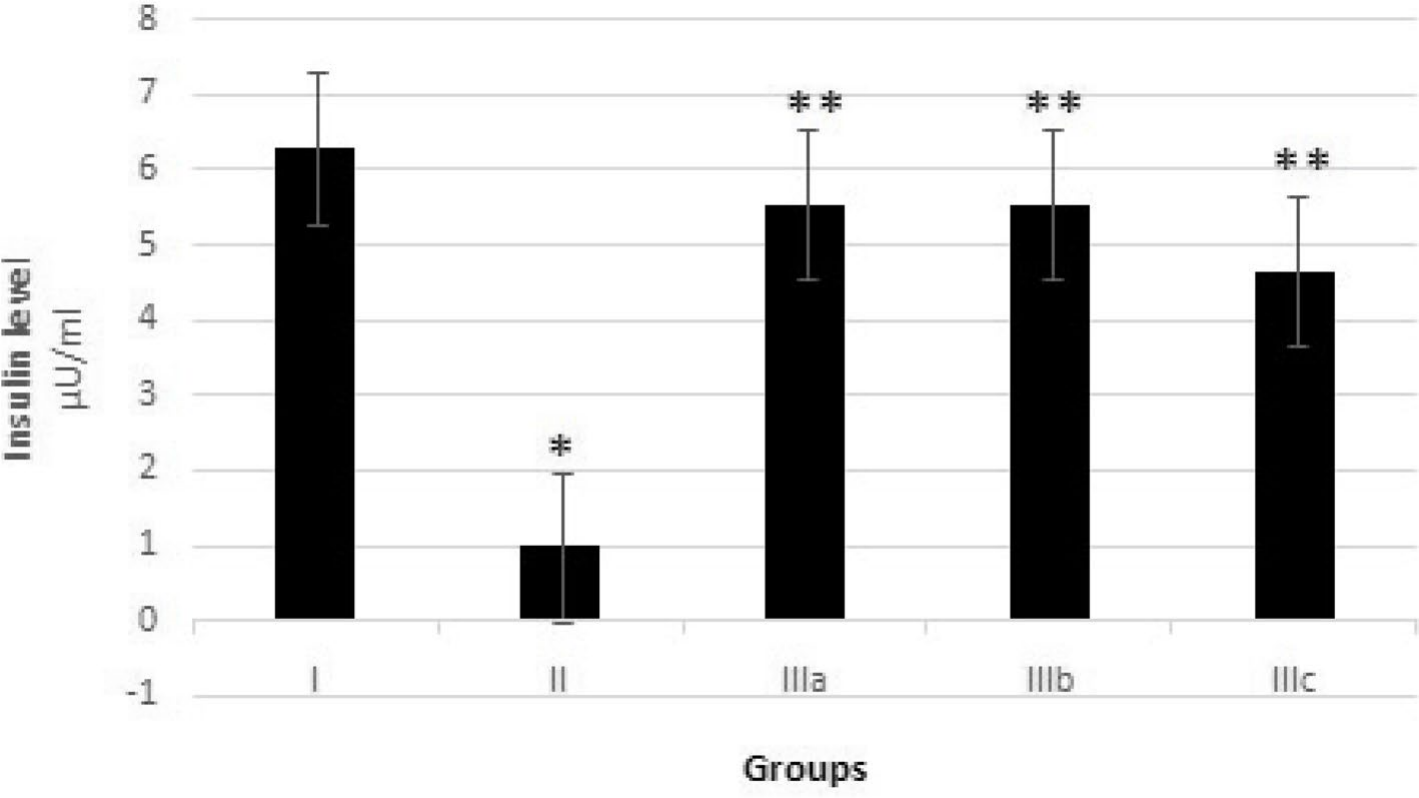
Mean serum insulin levels in different groups on the 30th day of experimental period. I. Control nondiabetic group; II. Diabetic group; IIIa. Fennel leaf aqueous extract 50 mg/kg BW; IIIb. Fennel leaf aqueous extract 100 mg/kg BW; IIIc. Fennel leaf aqueous extract 200 mg/kg BW.. * *P* < 0.05 significantly different from Group I; **P < 0.01 significantly different from Group II

The mean serum insulin level in the diabetic group decreased significantly (*P* < 0.05), as compared with the control animals. The mean serum insulin concentration of the diabetic rats that fennel leaf aqueous extract increased as compared with the diabetic animal rats 30th-day of experimental period. The serum insulin concentration of the diabetic rats that received fennel leaf aqueous extract at doses of 50, 100 and 200 mg/kg body weight was significantly higher than in the diabetic rats (*P* < 0.01) (Fig. 3).

### Effect of fennel leaf aqueous extract on blood C-peptide concentration

**Fig. 4:**
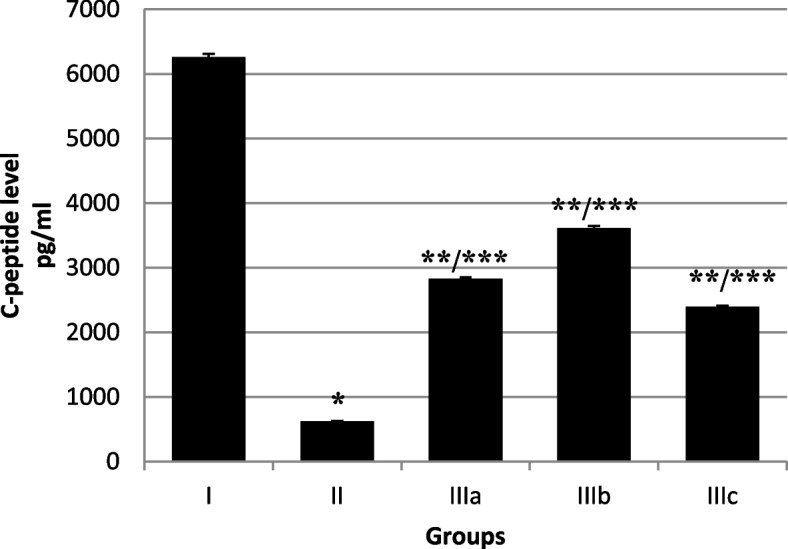
Mean blood C-peptide levels in different groups on the 30th day of experimental period. I. Control non-diabetic group; II. Diabetic group; IIIa. Fennel leaf aqueous extract 50 mg/kg BW; IIIb. Fennel leaf aqueous extract 100 mg/kg BW; IIIc. Fennel leaf aqueous extract 200 mg/kg BW. *P < 0.001, **P < 0.01 significantly different from Group I; ***P < 0.01significantly different from Group II

The mean blood C-peptide level in diabetic group decreased significantly (*P* < 0.001), compared with the non-diabetic control animals. The mean blood C-peptide concentration of the diabetic rats that received fennel leaf aqueous extract increased compared with the diabetic animals on 30thday of experimental period. The mean blood C-peptide concentration of the diabetic rats that received fennel leaf aqueous extract at doses of 50, 100 and 200 mg/kg body weight was significantly higher than that in the diabetic rats (*P* < 0.01) (Fig. 4).

### Histopathology of pancreatic islets

**Fig. 5:**
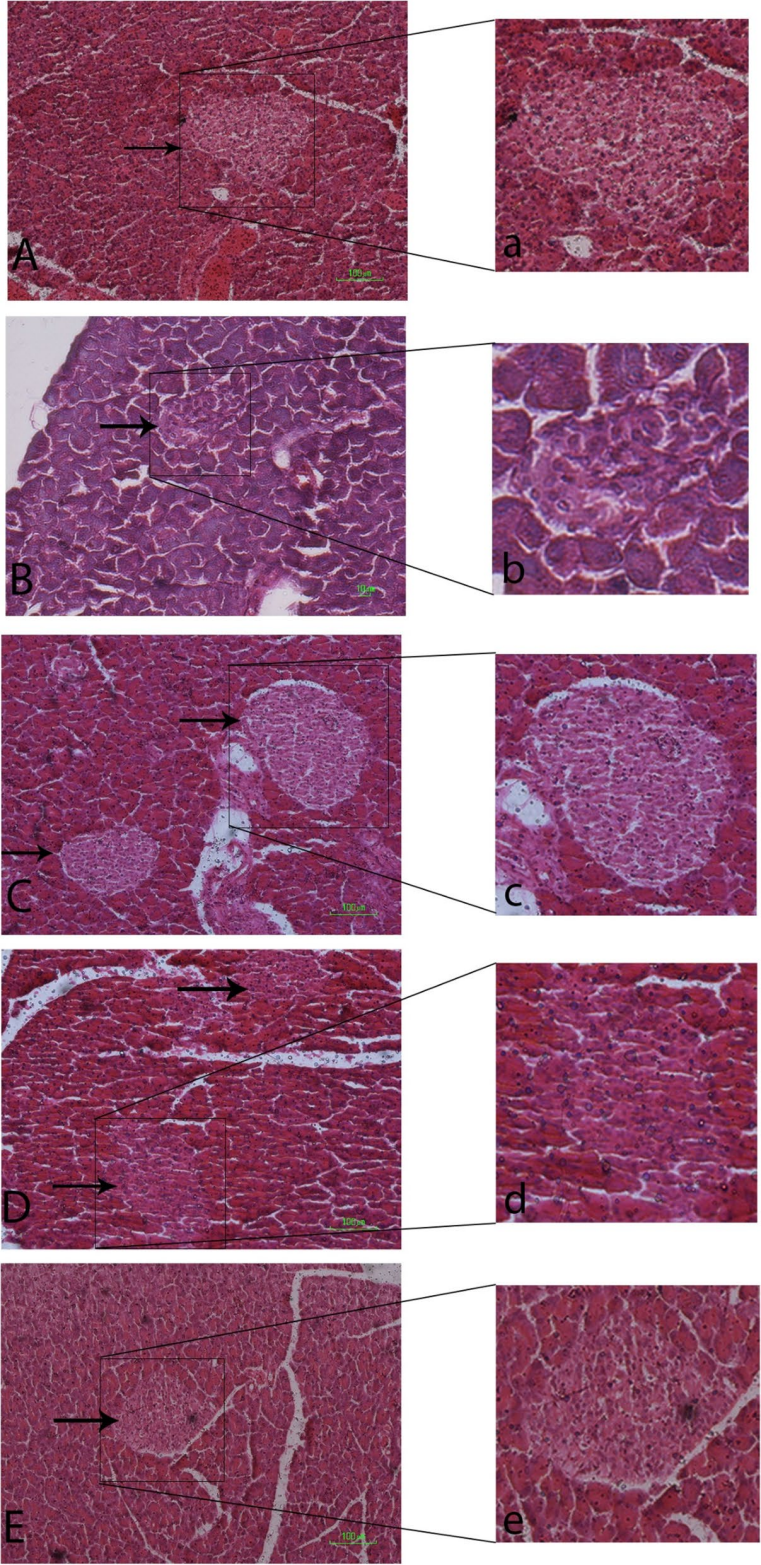
Sections of the pancreas stained by H&E. A,a. Section from a control normal rat (group I) showing a normal Islet (arrow); B,b. Section from the pancreas of a diabetic rat (group II) showing an atrophic appearance with irregular outlining of the islet (arrow); C,c; D,d; E,e. Sections from the pancreas of experimental groups IIIa, IIIb and IIIc presenting very similar morphology to the control normal group I with normal Islets (arrows)

The normal histology of the islets of Langerhans from the control group was shown in Fig. 5A. In the diabetic group, pancreatic islets were atrophied and detracted. Alloxan led to necrotic changes of the islets, especially in the center of the islets. The size and number of islets in the diabetic group obviously reduced (Fig. 5B).

**Fig. 6:**
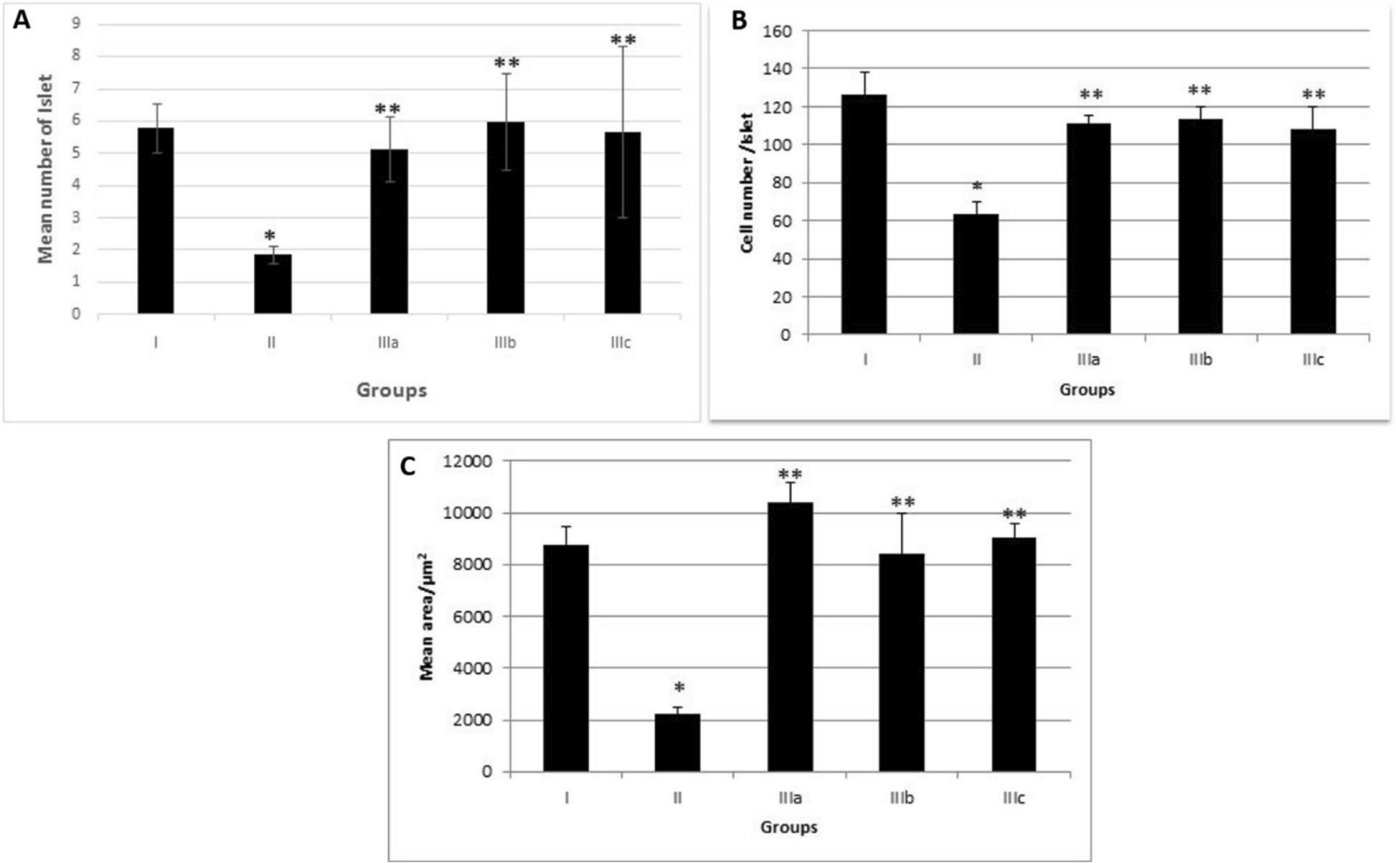
(**A**) Mean number of the islets of Langerhans in different groups at 30th-day of experimental period. *P < 0.05, significantly different from Group I; *P* < 0.05, significantly different from GroupII, (**B**). Mean cell number of the islets of Langerhans in different groups at 30th-day of experimental period. * *P* < 0.01, significantly different from Group I; ** *P* < 0.01, significantly different from GroupII. (**C**) Mean area of the islets of Langerhans in different groups at 30th-day of experimental period. * *P* < 0.01, significantly different from Group I; ** *P* < 0.01, significantly different from GroupII, I. Control nondiabetic group; II. Diabetic group; IIIa. Fennel leaf aqueous extract 50 mg/kg BW; IIIb. Fennel leaf aqueous extract 100 mg/kg BW; IIIc. Fennel leaf aqueous extract 200 mg/kg BW

Fennel leaf aqueous led to a protective effect in diabetic rats. In experimental groups, the pancreatic tissue retained its normal morphology (Figs. 5 and 6).

## Discussion

Present study showed fennel leaves aqueous extract can reduce blood glucose levels and intensity of weight loss, and increased serum insulin and C-peptide concentrations significantly; meanwhile morphological investigations showed that fennel leaf aqueous extract could induce significant improvements in morphology of pancreatic islets.

Diabetes mellitus is a common endocrine disorder characterized by hyperglycemia and the pathological changes in the pancreatic islets of Langerhans [19]. Most synthetic drugs have short- and long-term side effects, therefore herbal medicine is becoming increasingly interesting in the treatment of many disorders [20]. The WHO has urged that medicinal herbs be subsequently considered in the treatment of diabetes mellitus [21].

In the present study, biochemical and histopathological effects of fennel leaf aqueous extract were investigated on rats with alloxan-induced diabetes. Alloxan has been widely employed to induce type-1 diabetes in animal models. Alloxan is accessible, inexpensive and one of the most common agent for creation of type- 1 diabetes [22]. Injection of alloxan to the rats led to a reduction in insulin and then elevated serum glucose levels and weight loss.

Fennel is an aromatic plant with yellow flowers, feathery leaves and pharmacological properties. Although the impact of fennel essential oil on the hyperglycemia and pathological abnormalities in experimental diabetes was evaluated in many studies [13, 23], the experimental studies related to the leaf aqueous extract in diabetes are not well-documented. In traditional medicine, fennel tree leaves were recommended for the treatment of mouth ulcer, liver pain, and kidney ailments and diabetes mellitus [24].

The present biochemical findings supported the results of the previous studies, which reported that administration of fennel can lead to a decline of glucose levels, and the severity of weight loss compared to diabetic control group [23, 25].Topical and oral administration of fennel essential oil has shown to reduce glycemic levels [23]. Significant effects of fennel seeds extract on blood glucose were demonstrated [26].El-Ouadyet al described the leaves aqueous extract of fennel has a significant hypoglycemic effect in streptozotocin-induced diabetic rats [14]. The present study aligns with the findings of El-Ouady et al. [14], which showed that fennel leaf extract can reduce blood glucose levels. The main differences between our experiment and theirs include the diabetic model employed, variations in body weight, the geographical region where the fennel was cultivated, the dosage of fennel leaf extract used, the duration of extract administration, and the biochemical parameters measured, such as insulin and C-peptide, as well as histological evaluations. In our experiment, treatment with different doses (50,100 and 200 mg/kg body weight) of fennel leaf extract significantly decreased blood glucose.

Present study found that while 200 mg/kg of fennel leaf extract significantly reduced blood glucose levels, it had minimal impact on body weight. Some evidence supports that higher doses of fennel leaf extract may influence body weight changes, as fennel has been shown to decrease food efficiency [27] and manage appetite [28]. However, other studies indicate that fennel does not affect body weight [14, 29] and [30]. Therefore, it is possible that higher doses of the extract could prevent weight gain.

The identified ingredients of fennel leaf such asphenolic acids (caffeic acid, quinic acid, and chlorogenic acid) have been exhibited to reduce blood glucose and anti-diabetic activity [9]. Caffeic acid reduces blood glucose and reveals a protective effect against diabetes [31]; quinic acid increases mitochondrial Ca^2+^ and stimulate insulin secretion in pancreatic beta‐cells [32]; chlorogenic acid has a protective effect on pancreatic beta‐cells and blood glucose control [33]. It seems different reasons are responsible for reducing blood-glucose such as: raise the insulin sensitivity of the receptors on cells, changes in energy metabolism and increases in insulin secretion from remaining pancreatic cells, increasing absorption of dietary carbohydrates in alimentary tract or facilitation of glucose consumption by peripheral tissues [34].

Our histological findings showed that fennel leaf extract reduced islets of Langerhans damage in diabetic rats. In diabetic animals, pancreatic β-cells were destroyed and their number declined. This causes a reduction in serum insulin concentration. In our experiment, we considered the effect of fennel leaf aqueous extract on the histopathology of pancreas and the investigation of islets of Langerhans in rats with alloxan-induced diabetes. Our findings showed fennel leaf aqueous extract preserved the size and number of the islets following induction of diabetes. It seems fennel leaf extract can protect the islets against alloxan toxicity. Our results confirm the report of protective effects fennel essential oil on pancreatic islets [25]. The oxidative stress due to hyperglycemia led to the histopathological changes in pancreas gland [35, 36]. Diabetes elevates free radical generation and accordingly decrease antioxidant levels [35]. It seems anti-oxidative effects of fennel improves the changes and carried out production of insulin as a result of pancreatic exocrine and endocrine regeneration [25]. It can be explained that phenolic compounds of fennel underlie the anti-oxidative effects and the regenerative capability of fennel [37].

Our research suggests that regardless of insulin levels, higher amounts of fennel leaf extract do not lead to increased C-peptide levels compared to lower amounts. This result could be associated with nephropathy observed in diabetic animals. While insulin is primarily processed by the liver, C-peptide is mainly metabolized by the kidneys [38]. Because of the potential for diabetes-related neuropathy or the effects of a high dose of fennel leaf extract, there may be an imbalance between insulin and C-peptide ratios. More research is necessary to verify this hypothesis.

## Conclusion

We conclude that the administration of fennel leaf aqueous extract improved the hyperglycemia and histopathological changes of the pancreatic islets of Langerhans. The present findings may support the therapeutic effects of fennel leaf extract as complementary treatments in diabetes.

## Data Availability

The data that support the findings of this study are available from the corresponding author upon reasonable request.
